# SR-BI Interactome Analysis Reveals a Proviral Role for UGGT1 in Hepatitis C Virus Entry

**DOI:** 10.3389/fmicb.2019.02043

**Published:** 2019-09-06

**Authors:** Jiazhao Huang, Han Yin, Peiqi Yin, Xia Jian, Siqi Song, Junwen Luan, Leiliang Zhang

**Affiliations:** ^1^Institute of Basic Medicine, Shandong First Medical University & Shandong Academy of Medical Sciences, Jinan, China; ^2^NHC Key Laboratory of Systems Biology of Pathogens, Institute of Pathogen Biology, Chinese Academy of Medical Sciences & Peking Union Medical College, Beijing, China

**Keywords:** HCV, SR-BI, UGGT1, calnexin, N-glycosylation

## Abstract

Hepatitis C virus (HCV) entry is mediated by multiple co-receptors including scavenger receptor class B, type I (SR-BI). To elucidate the interactome of human SR-BI, we performed immunoprecipitation (IP) experiment coupled with mass spectrometry (MS) analysis. UDP-glucose:glycoprotein glucosyltransferase 1 (UGGT1), a key component of calnexin cycle involved in protein glycosylation, was identified as a SR-BI-interacting protein. Silencing UGGT1 or N-glycosylation inhibitor treatment reduced SR-BI protein level. Further study demonstrated that human SR-BI was N-glycosylated at nine asparagines. Moreover, HCV entry and infection were reduced by the absence of UGGT1. Interestingly, silencing SR-BI reduced protein stability of UGGT1 and protein quality control function mediated by UGGT1. Our finding not only identified UGGT1 as a HCV host factor, but also identified a UGGT1-mediated protein folding function for SR-BI.

## Introduction

Hepatitis C virus is an enveloped positive-stranded RNA virus belonging to the *Hepacivirus* genus in the *Flaviviridae* family (Lindenbach and Rice, 2003). Currently, no proved vaccine against HCV is available. It is estimated that HCV chronically infected more than 71 million people worldwide, despite the revolutionized hepatitis C management of direct-acting antiviral agents development in recent years (Gotte and Feld, 2016; Martinello et al., 2018). HCV infection consequently increased the risk of developing liver diseases, such as cirrhosis and hepatocellular carcinoma.

Following HCV attachment, the viral particle interacts with important entry co-receptors, such as CD81, claudin-1, occludin, and scavenger receptor class B, type I (SR-BI) (Zeisel et al., 2013; Ding et al., 2014). SR-BI interacts with HCV E2 to mediate HCV entry (Scarselli et al., 2002) and is shown to be a promising anti-HCV target. Anti-SR-BI monoclonal antibodies and a small inhibitor against SR-BI have been found to inhibit HCV infection (Colpitts and Baumert, 2016). SR-BI is also a key receptor for high density lipoproteins (HDL), which mediates selective uptake of HDL-cholesteryl esters into the liver (Acton et al., 1996). Recent study found that residue 175 of human SR-BI is part of an N-linked glycosylation sequence (NXS/T) (Brunham et al., 2011; Chadwick and Sahoo, 2012). Mutation of Thr175 to Ala disrupts proper glycosylation of Asn173 (Chadwick and Sahoo, 2012). T175A mutant of SR-BI did not support HCV entry (Westhaus et al., 2017). Mouse SR-BI contains 11 N-glycosylation sites (Vinals et al., 2003). However, human N-glycosylation sites in human SR-BI remain poorly defined.

Glycoproteins were synthesized in the endoplasmic reticulum (ER), where their folding is surveyed by the UDP-glucose:glycoprotein glucosyltransferase 1 (UGGT1) (Christianson et al., 2011; Hebert and Molinari, 2012). This enzyme acts as a glycoprotein folding quality control checkpoint, which selectively reglucosylates misfolded glycoproteins, promotes their association with calnexin/calreticulin chaperone system, and prevents premature secretion from the ER (Hebert and Molinari, 2012). UGGT1 recognizes hydrophobic or disordered patches near asparagine-linked non-glycosylated glycans in partially misfolded glycoproteins and reglucosylates them, returning folding intermediates to the calnexin/calreticulin cycle (Hebert and Molinari, 2012).

To obtain a more complete picture of interaction partners of SR-BI and to predict function, we applied immunoprecipitation (IP) coupled with a mass spectrometry (MS) based approach. 28 proteins were identified that potentially interacted with SR-BI. One of novel hits was UGGT1. UGGT1 functioned in N-glycosylation of SR-BI as a sensor for protein quality control. Silencing UGGT1 reduced protein level of SR-BI and HCV entry. Interestingly, we found that SR-BI stabilized UGGT1. Silencing SR-BI resulted in reduction of UGGT1 protein and disrupted the UGGT1-mediated folding of N-glycosylated protein. Our results therefore identify UGGT1 as a player in HCV infection, and provide a mechanism to explain the migration role of SR-BI.

## Materials and Methods

### Cells, Virus, and Reagents

293T and Huh7.5.1 cells were maintained in Dulbecco’s modified Eagle’s medium (DMEM, Thermo Fisher Scientific, Waltham, MA, United States) supplemented with 10% fetal bovine serum (FBS), glutamine and gentamicin. Jc1Flag (p7-nsGluc2A) was obtained from Dr. Charles Rice (Jones et al., 2010). For Jc1Flag (p7-nsGluc2A) virus infection, Huh7.5.1 cells were incubated with Jc1Flag (p7-nsGluc2A) virus in DMEM with 2% FBS for 6 h. Then the inoculum was removed and the cells were incubated in DMEM with 10% FBA. HCV pseudovirus particles (HCVpp) or VSV pseudovirus particles (VSVpp) were produced in 293T cells by transfection with plasmids encoding HIV Gag/Pol (pLP1), HIV Rev (pLP2), pLenti6 encoding luciferase, and HCV E1E2 from genotype 2a strain or the VSV-G expression plasmid (provided by Dr. Ping Zhao) (Cao et al., 2011; Guan et al., 2012). Tunicamycin was from abcam (Cambridge, MA, United States, catalog No. ab120296). PNGase F was from NEB (Ipswich, MA, United States, catalog No. P0704S).

### Plasmids

The constructs encoding SR-BI-Flag, CD81-Flag, occludin-Flag, and claudin1-Flag were inserted into p3xFlag-CMV-14 vector. The construct expressing calnexin-Flag is pECMV-3 × FLAG-CANX from Miaoling (Wuhan, China). The constructs encoding SR-BI-Flag mutant N2Q, N4Q, N5Q, N6Q, N7Q, N8Q, N9Q, and N9D were generated using a QuikChange site-directed mutagenesis kit. Construct encoding Flag-SR-BI was inserted into the PCMV-Tag2 vector. UGGT1-Flag is a gift from Dr. Shin-Ru Shih (Huang et al., 2017). NHK-GFP is a gift from Dr. Ron R Kopito (Christianson et al., 2011).

### Antibodies

The following primary mouse antibodies were used: anti-actin (Sigma-Aldrich, St. Louis, MO, United States, catalog No. A2228), anti-Flag (Sigma-Aldrich, St. Louis, MO, United States, catalog No. A2220), IgG control (MBL, Nagoya, Japan, catalog No. M075-3), anti-SR-BI (BD Biosciences, Franklin Lakes, NJ, United States, catalog No. 610883), and anti-UGGT1 (Santa Cruz Biotechnology, Santa Cruz, CA, United States, catalog No. Sc-374565). The following primary rabbit antibodies were used: anti-calnexin (Cell Signaling Technology, Boston, MA, United States, catalog No. 2433), anti-SR-BI (Novus Biological, Centennial, CO, United States, catalog No. NB400-104). The secondary antibodies included: HRP-conjugated ECL goat anti-rabbit IgG (Sigma-Aldrich, St. Louis, MO, United States, catalog No. A6154), HRP-conjugated ECL goat anti-mouse IgG (Sigma-Aldrich, St. Louis, MO, United States, catalog No. A4416), donkey anti-mouse-Alexa Fluor 488, donkey anti-rabbit-Alexa Fluor 594, donkey anti-rabbit-Alexa Fluor 488, donkey anti-mouse-Alexa Fluor 594, donkey anti-rat-Alexa Fluor 488 (Invitrogen, Carlsbad, CA, United States).

### Immunoprecipitation

The IP experiment has been described previously (Yin et al., 2016). Briefly, cells were lysed with lysis buffer (1% Triton X-100, 50 mM Tris–HCL, pH 7.4, 150 mM NaCl, and protease inhibitor cocktail) and precleared by the addition of protein A/G beads for 30 min at 4°C. The lysates were then incubated with protein A/G beads prebound with antibody for 1.5 h at 4°C. The samples were washed three times with PBS, eluted in SDS sample buffer, and analyzed by Western blotting.

### Mass Spectrometry

To identify SR-BI-interacting proteins, lysates from 293T cells transfected with Flag-SR-BI, or Flag vector control were immunoprecipited by Flag antibody. The interacting proteins were analyzed by electrospray ionization tandem MS on a Thermo LTQ Orbitrap instrument. Proteins identified from the SR-BI-Flag but not from the p3xFlag-CMV-14 control sample were detected. Individual ion scores are indicated in the form of a Mascot-derived confidence score [calculated from the posterior error probability (PEP) as 10 log(PEP)]. The default significance threshold is a *P*-value of <0.05.

### Immunofluorescence Microscopy

Immunofluorescence microscopy has been described previously (Yin et al., 2017). Briefly, cells seeded onto glass coverslips were washed with PBS and fixed with methanol (−20°C) for 5 min at room temperature. Fixed cells were washed with PBS three times and then incubated with blocking solution (PBS containing 10% normal donkey serum) for 5 min at room temperature. Next, the coverslips were incubated with primary antibodies in permeabilizing buffer (0.5% Triton X-100 in PBS containing 10% normal donkey serum) for 1 h. The coverslips were then washed three times with blocking solution, followed by incubation with Alexa Fluor 488- and Alexa Fluor 555-conjugated secondary antibodies for 1 h at room temperature. After being washed three times with blocking solution, the coverslips were mounted with mounting medium containing 4′,6-diamidino-2-phenylindole (DAPI). The cells were imaged using a Leica TCS SP5 microscope (Germany) equipped with a 40X oil immersion lens.

### Knockdown by siRNA

siRNAs were transfected into cells using Lipofectamine^TM^ RNAiMAX Transfection Reagent (Invitrogen, Carlsbad, CA, United States). For a 24-well plate, 40 μl Opti-MEM was incubated with 0.8 μl of Lipofectamine^TM^RNAiMAX, and 1.25 μl siRNA was mixed with 40 μl Opti-MEM in separate sterile 1.5 ml tubes. Then, the two tubes were mixed and allowed to stay at room temperature for about 15 min. Digest the cells, and RNAi mixture, cell suspension and medium were sequentially added to the 24-well plate. The siRNAs were from GenePharma (Shanghai, China): UGGT1 siRNA#1: GCUGUGAGCUCAGAACUUATT; UGGT1 siRNA#2: GGUCAUUGCUACGACAUCATT; SR-BI siRNA#1: CAAGUUCGGAUUAUUUGCUTT; SR-BI siRNA#2: CAUGAUCAAUGGAACUUCUTT; UGGT2 siRNA: GCCUU UGAAAGUCUGGGAATT; control siRNA#1: ACGUGACACG UUCGGAGAATT; control siRNA#2: GCGACGAUCUGCCU AAGAUdTdT.

### Reporter Assay

Hepatitis C virus infection was assessed by monitoring the *Gaussia* luciferase activity in Jc1Flag2(p7-nsGluc2A)-infected Huh7.5.1 cells (Promega, Madison, WI, United States). HCVpp- or VSVpp-infected Huh 7.5.1 cells were assessed by monitoring the firefly luciferase activity (Promega, Madison, WI, United States). Cell viability was determined by measuring the cellular ATP level using a CellTiter-Glo luminescent cell viability assay kit (Promega, Madison, WI, United States) according to the manufacturer’s protocol. The normalized luciferase activity was determined by dividing the luciferase activity by the ATP level.

### PNGase F Treatment

1–20 μg of cell lysates, 1 μl of Glycoprotein Denaturing Buffer (10X) and H2O (if necessary) were combined to make a 10 μl total reaction volume. Samples were denatured by heating reaction at 100°C for 10 min and then chilled on ice and centrifuged for 10 s. A total reaction volume of 20 μl was made by adding 2 μl GlycoBuffer 2 (10X), 2 μl 10% NP-40 and 6 μl H2O. 1 μl PNGase F was added to the reaction system and mixed gently. The reaction system was incubated at 37°C for 1 h and analyzed by SDS-PAGE followed by Western blotting.

### Protein Folding Assay

Protein folding assay has performed according the reference (Chen et al., 2017). Briefly, cells were treated with siRNAs for 48 h and then transfected with construct expressing NHK-GFP, a GFP-fused version of the NHK folding variant of 1-antitrypsin for another 24 h. Cells were lysed with NP-40-containing lysis buffer (50 mM Tris–HCl pH 7.4, 150 mM NaCl, 5 mM EDTA, and 0.2% NP-40, supplemented with protease inhibitor) for 1 h on ice. Lysates were then centrifuged at 13,500 *g* for 15 min at 4°C to separate insoluble portion (pellet) and soluble portion (supernatant); the former were washed twice with lysis buffer containing 0.1% NP-40 and material solubilized by 95°C incubation for 10 min in SDS-containing sample buffer (50 mM Tris, 4% SDS, 4% 2-mercaptoethanol, 12% glycerol) prior to SDS-PAGE followed by Western blotting.

### Quantitative PCR (qPCR)

Cellular RNA was isolated using TRIzol reagent (Invitrogen, CA, United States) and reverse transcribed with the High Capacity cDNA Reverse Transcription Kit (Applied Biosystems; Foster City, CA, United States), then quantitated by qPCR using the DyNAmo HS SYBR Green qPCR Kit (Finnzyme; Espoo, Finland). Sequences of primers used in qPCR were as follows: The forward and reverse primers for GAPDH were 5′-GAAGGTGAAGGTCGGAGTC-3′ and 5′-GAAGATGGTGATGGGATTTC-3′; the forward and reverse primers for UGGT1 were 5′-GCCTTCCAGCAGATAGCAGC-3′ and 5′-GCTTTCAGGATTAGACGAGGGAT-3′;the forward and reverse primers for SR-BI were 5′-AGCCGCTCGAA GTATGCAC-3′ and 5′-CTTGCCACAGGACACCTTCA-3′.

### Statistics

Statistically significant differences were assessed using the paired Student’s *t*-test in GraphPad Prism 5 (GraphPad Software Inc., La Jolla, CA, United States). Unless otherwise stated, the data represent the means from at least three independent experiments ± the standard deviation (SD). ^∗∗^*P* < 0.01; ^∗∗∗^*P* < 0.001; ns, not significant.

## Results

### Interactome Analysis of SR-BI Identified the Interaction Between SR-BI and Calnexin Cycle Components

**FIGURE 1:**
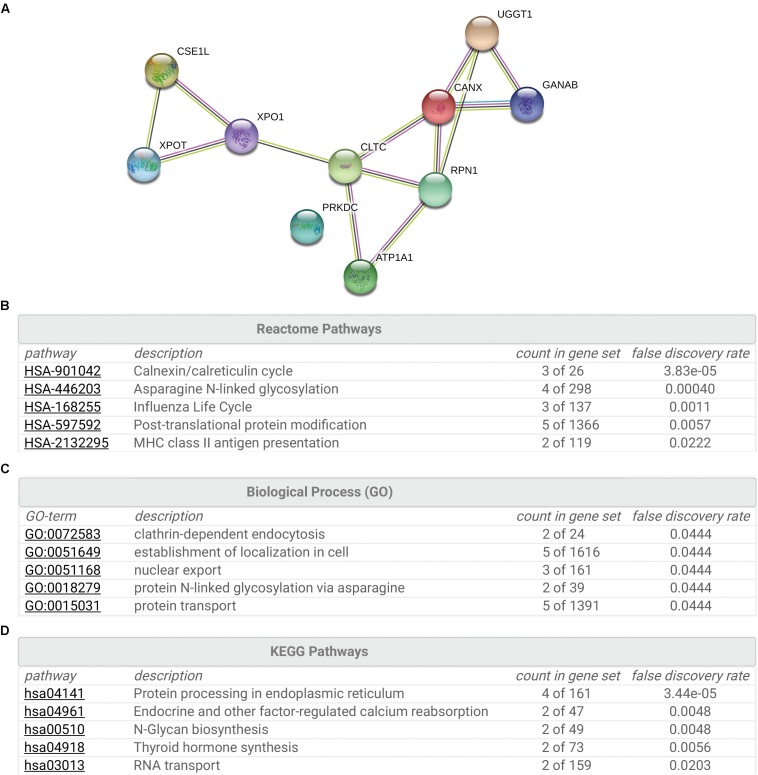
Interactome analysis of SR-BI. **(A)** String analysis showed top 10 proteins that interacted with SR-BI from LC-MS/MS experiments. **(B–D)** Reactome Pathways **(B)**, Biological Process **(C)** and KEGG Pathway **(D)** analysis of the top 10 potential binding partners of SR-BI.

To gain insight into the cellular functions of SR-BI, we analyzed the proteins that potentially interacted with SR-BI. Firstly, we performed IP experiments with Flag antibody using 293T cells transfected with plasmids expressing Flag-SR-BI, or Flag vector control. Next, the samples were analyzed by MS. Supplementary Table S1 was the list of the top 28 SR-BI interactors, whose scores were above 500. String analysis of the top 10 proteins identified calnexin cycle components including UGGT1 and calnexin as putative SR-BI binding partners (Figures 1A,B). Biological Process and KEGG Pathway analysis of the top 10 hits indicated that UGGT1 and calnexin are involved in the N-glycosylation process (Figures 1C,D).

### SR-BI Associated With UGGT1 and Calnexin

**FIGURE 2:**
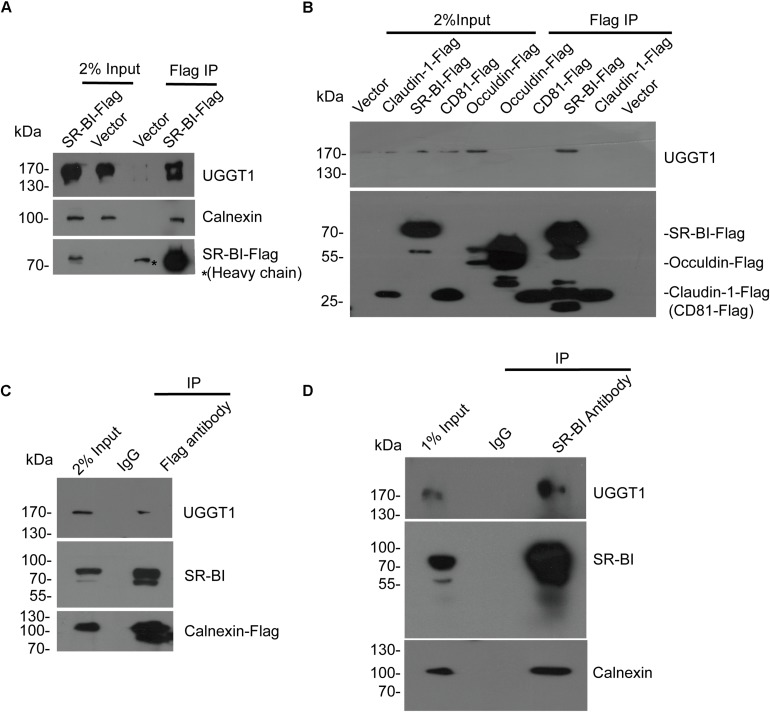
Validation of the interactions between SR-BI and components of calnexin cycle. **(A)** Co-immunoprecipitation assay showed SR-BI-Flag could interact with UGGT1 and calnexin. SR-BI-Flag or p3xFlag-CMV-14 empty construct was transfected into 293T cells for 48 h. The cell lysates were incubated with anti-Flag antibody-coated beads and co-IP samples were subjected to SDS-PAGE followed by Western blotting with indicated antibodies. ^∗^ marked antibody heavy chain. **(B)** UGGT1 interacted with SR-BI but not other HCV receptors. Four constructs expressing C-termed Flag tagged HCV receptors or p3xFlag-CMV-14 empty vectors were transfected into 293T cells for 48 h. Cell lysates were incubated with anti-Flag antibody-coated beads and detected by SDS-PAGE followed by Western blotting with indicated antibodies. **(C)** Co-immunoprecipitation assay showed calnexin interacted with SR-BI and UGGT1. Calnexin-Flag construct was transfected into 293T cells for 48 h and then cell lysates were incubated with anti-Flag antibody or control mouse IgG -coated beads. Co-IP samples were subjected to SDS-PAGE followed by Western blotting with indicated antibodies. **(D)** Co-immunoprecipitation assay of endogenous SR-BI showed SR-BI interacted with UGGT1 and calnexin. Huh7.5.1 cell lysates were incubated with anti-SR-BI antibody or control mouse IgG-coated beads. Co-IP samples were subjected to SDS-PAGE followed by Western blotting with indicated antibodies.

To validate that UGGT1 and calnexin are SR-BI interactors, we performed further co-immunoprecipitation experiments. SR-BI-Flag and p3xFlag-CMV-14 empty vector were transfected into 293T cells for 48 h. The cells were lysed by the IP buffer, and then were incubated with anti-Flag antibody-coated beads and Co-IP proteins were subjected to Western blotting for analysis. We found SR-BI-Flag was precipitated successfully as well as cellular UGGT1 and calnexin (Figure 2A).

To confirm the specificity of UGGT1/SR-BI interaction, four C-terminal Flag-tagged HCV receptors (CD81, occludin, SR-BI, and claudin-1) and p3xFlag-CMV-14 were individually transfected into 100 mm plate of 293T cells for 48 h. The cell lysates were incubated with Flag antibody for IP followed by Western blotting analysis. Among those HCV receptors, UGGT1 only associated with SR-BI but not other HCV-receptors (Figure 2B).

Next, calnexin-Flag was transfected into 293T cells, and cell lysates were incubated with anti-Flag antibody and IgG-coated beads. SR-BI and UGGT1 were detected in the Flag IP condition but not in IgG IP condition, suggesting that SR-BI and UGGT1 interacted with calnexin (Figure 2C). To further confirm that endogenous SR-BI interacted with UGGT1 and calnexin, Huh7.5.1 were lysed by IP buffer and then incubated with anti-SR-BI antibody and IgG-coated beads and Co-IP samples were subjected to Western blotting analysis. As shown in Figure 2D, endogenous SR-BI could associate with UGGT1 and calnexin.

**FIGURE 3:**
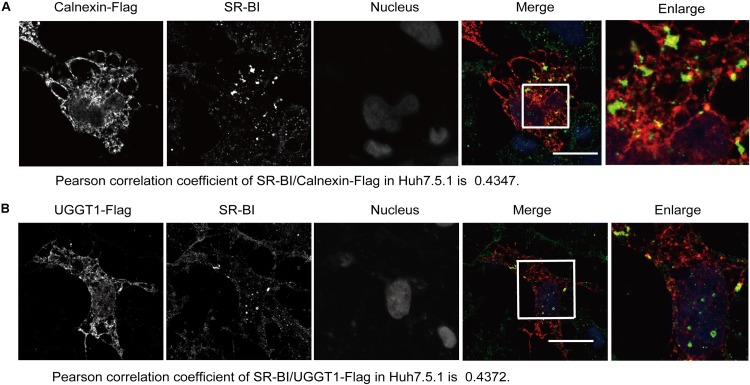
Scavenger receptor class B, type I partially colocalized with UGGT1 and calnexin. **(A)** Colocalization of SR-BI with calnexin-Flag in Huh7.5.1 cells. Huh7.5.1 cells transfected with calnexin-Flag construct were fixed by methanol, permealized by 0.5% Triton X-100 and immunofluorescently labeled for SR-BI antibody (green) and Flag antibody (red). DAPI marked nucleus (blue). Scale bar, 15 μm. **(B)** Colocalization of SR-BI with UGGT1-Flag in Huh7.5.1 cells. Huh7.5.1 cells transfected with UGGT1-Flag construct were fixed by methanol, permealized by 0.5% Triton X-100 and immunofluorescently labeled for SR-BI antibody (green) and Flag antibody (red). DAPI marked nucleus (blue). Scale bar, 15 μm.

The specific interaction of SR-BI with the UGGT1 and calnexin promoted us to examine whether these proteins colocalized within the cells. Huh7.5.1 cells transfected calnexin-Flag were fixed by methanol, permealized by 0.5% Triton X-100 and immunofluorescently labeled. Immunofluorescence staining showed that both UGGT1 and calnexin were present in the ER (Figures 3A,B). SR-BI (green) and Calnexin-Flag (red) has partial colocalization in Huh7.5.1 cells when their respective images were merged (Figure 3A). Partial co-localization of SR-BI and UGGT1-Flag in Huh7.5.1 cells was also detected by the same method as above (Figure 3B). Taken together, SR-BI and UGGT1/calnexin were partially present in the same subcellular compartment.

### Protein Level of SR-BI Was Dependent on UGGT1 and N-Glycosylation of SR-BI

**FIGURE 4:**
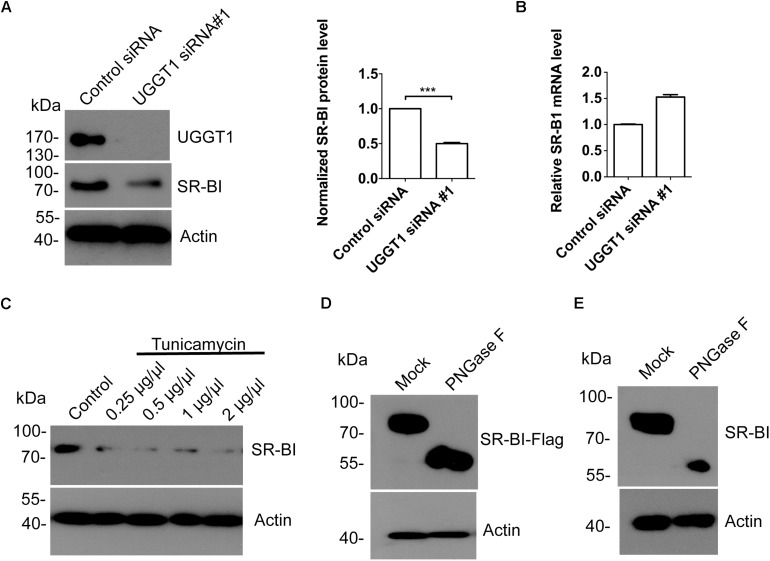
Silencing UGGT1 or tunicamycin treatment reduced protein level of SR-BI. **(A)** Silencing UGGT1 reduced SR-BI protein quantity. Huh7.5.1 cells were treated with UGGT1 siRNA#1or control siRNA for 72 h and then cell lysates were subjected to SDS-PAGE followed by Western blotting with indicated antibodies. The blots of three independent experiments were quantified using ImageJ. Relative protein level of SR-BI was normalized to the blot signal of actin. **(B)** Huh7.5.1 cells were treated with siRNA targeting UGGT1 or control siRNA for 72 h. Total RNA was isolated and reverse transcribed and then the qPCR was performed. Relative SR-BI mRNA level was normalized to GAPDH mRNA level. **(C)** Protein level of SR-BI was decreased by tunicamycin, an inhibitor of N-glycosylation. Huh7.5.1 cells were treated with different dosages of tunicamycin for 16 h and then the cell lysates were subjected to SDS-PAGE followed by Western blotting with indicated antibodies. **(D)** PNGase F reduced the size of SR-BI-Flag. Huh7.5.1 cells transfected with SR-BI construct were treated with PNGase F or mock treatment. Then cell lysates were subjected to SDS-PAGE followed by Western blotting with indicated antibodies. **(E)** Size of endogenous SR-BI was decreased by PNGase F. Cell lysates from Huh7.5.1 cells treated with PNGase F or mock treatment were subjected to SDS-PAGE followed by Western blotting with indicated antibodies. ^∗∗∗^*P* < 0.001.

To explore the relationship between SR-BI and UGGT1, we knocked down UGGT1 in Huh7.5.1 cell using UGGT1 siRNA#1 and control siRNA for 72 h. Western blotting of cell lysates showed that silencing UGGT1 decreased SR-BI protein level (Figure 4A). However, the mRNA level of SR-BI was not reduced by silencing UGGT1 (Figure 4B). UGGT1 was an important glycosyltransferase, thus we hypothesized that UGGT1 may facilitate SR-BI glycosylation. SR-BI quantity decreased may be due to mis-glycosylated SR-BI degradation. To test this, we treated Huh7.5.1 cells with different concentrations of tunicamycin, which was an inhibitor for N-glycosylation. Protein level of SR-BI was decreased in the presence of tunicamycin compared with control treatment (Figure 4C). PNGase F is an enzyme to catalyze de-N-glycosylation. 293T cells lysates transfected with SR-BI-Flag were treated with PNGase F and detected by Western blotting using Flag antibody. As shown in Figure 4D, the size of SR-BI-Flag was decreased from about 90 kd to about 60 kd in the condition of PNGase F, indicating the N-glycosylation modification of SR-BI. Furthermore, glycosylated bands of endogenous SR-BI vanished after PNGase F treatment, suggesting that these bands were N-glycosylated SR-BI (Figure 4E). Together, those data suggested that UGGT1 might be involved in N-glycosylation and proper folding of SR-BI.

### All Nine Asparagines of Predicted SR-BI N-Glycosylation Sites Participated in SR-BI N-Glycosylation

**FIGURE 5:**
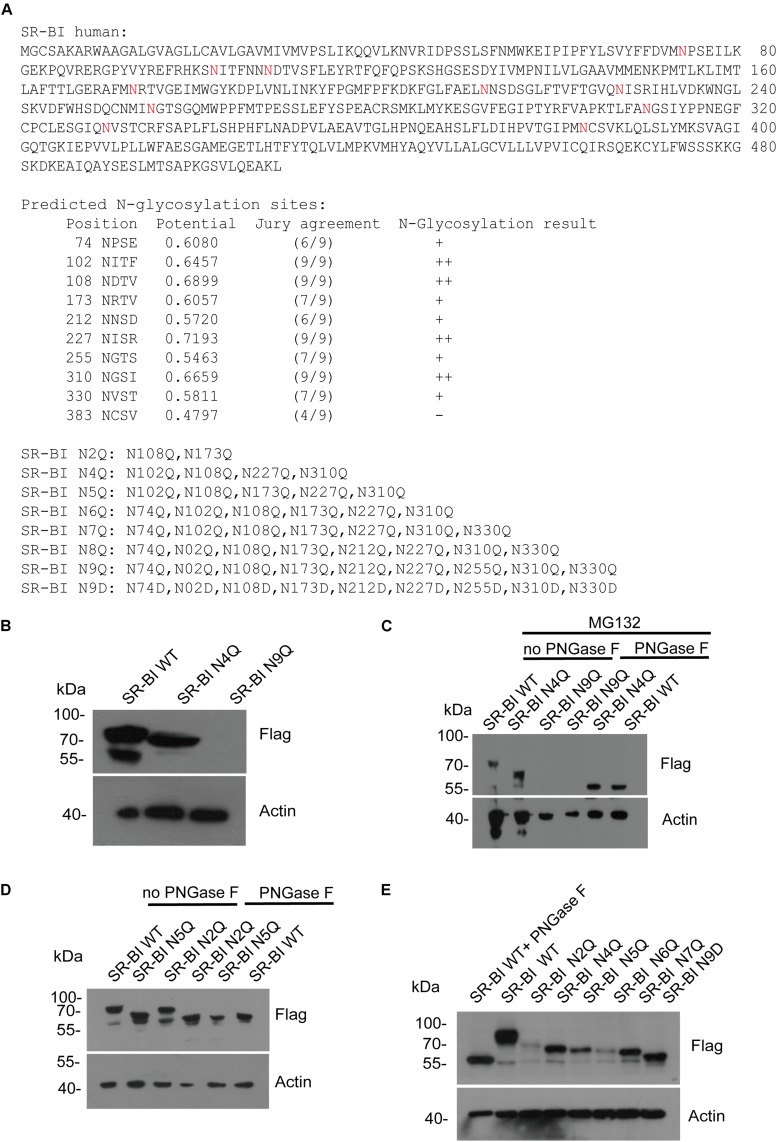
Human SR-BI was N-glycosylated. **(A)** Prediction of potential N-glycosylation site in human SR-BI. Human SR-BI sequence was predicted through N-Gly website (http://www.cbs.dtu.dk/services/NetNGlyc/). The potential N-glycosylation sites of human SR-BI was labeled in red. SR-BI constructs with N to Q or D mutation were shown. **(B)** SR-BI was decreased when four N were mutated to Q(N4Q). 293T cells were transfected with constructs expressing Flag tagged SR-BI-WT, SR-BI N4Q, SR-BI N9Q for 48 h and then cell lysates were subjected to SDS-PAGE followed by Western blotting with indicated antibodies. **(C)** Size of SR-BI of N4Q was reduced by PNGase F in the presence of MG132. 293T cells were transfected with constructs expressing Flag tagged SR-BI-WT, SR-BI N4Q, SR-BI N9Q for 48 h and MG132 was presence in the last 12 h. Cell lysates were treated with PNGase F or mock treatment and then subjected to SDS-PAGE followed by Western blotting with indicated antibodies. **(D)** Size of SR-BI of N2Q mutant was between N5Q and WT while PNGase F decreased all of them to the same size. 293T cells were transfected with constructs expressing Flag tagged SR-BI-WT, SR-BI N2Q, or SR-BI N5Q for 48 h and cell lysates were treated with PNGase F or mock treatment. The samples were subjected to SDS-PAGE followed by Western blotting with indicated antibodies. **(E)** Size of SR-BI became smaller with increased mutation sites. 293T cells were transfected with constructs expressing Flag tagged SR-BI-WT, SR-BI N2Q, SR-BI N4Q, SR-BI N5Q, SR-BI N6Q, SR-BI N7Q, or SR-BI N9D for 48 h and cell lysates were subjected to SDS-PAGE followed by Western blotting with indicated antibodies.

Next, we predicted the potential N-glycosylation sites of human SR-BI through N-Gly website^1^. Nine sites (N in red) were identified as shown in Figure 5A. Based on this information, we constructed seven mutants of human SR-BI (Figure 5A). As shown in Figure 4B, SR-BI protein size was decreased when four most potential N-glycosylated N were mutated to Q (N4Q). Interestingly, SR-BI protein was undetectable when all nine potential N-Gly sites were mutated to Q (N9Q), indicating that N9Q of SR-BI did not express well. Mutation of N4Q appeared to disrupt N-glycosylation residues as judged by its faster migration as compared to the wild-type SR-BI in SDS-PAGE (Figure 5B). Glycosylated bands vanished after PNGase F treatment, suggesting that these bands were N-glycosylated SR-BI (Figure 5C). Size of SR-BI of N4Q mutant was still larger than de-N-glycosylated SR-BI generated by PNGase F treatment (Figure 5C). Size of SR-BI N2Q mutant was between N5Q and WT. PNGase F treatment could make N5Q, N2Q and WT decreased to de-N-glycosylation SR-BI size (Figure 5D). Since SR-BI N9Q could not express well, we generated SR-BI N9D mutant (Figure 5A). It should be noticed that we did not detect the expression of SR-BI N8Q. When we compared the protein sizes of SR-BI WT and SR-BI mutants, the more sites in SR-BI were mutated, the smaller SR-BI size was (Figure 5E), suggesting that all nine potential N-Gly sites were involved in SR-BI N-glycosylation process.

### UGGT1 Influenced SR-BI Level and Promoted HCV Infection

**FIGURE 6:**
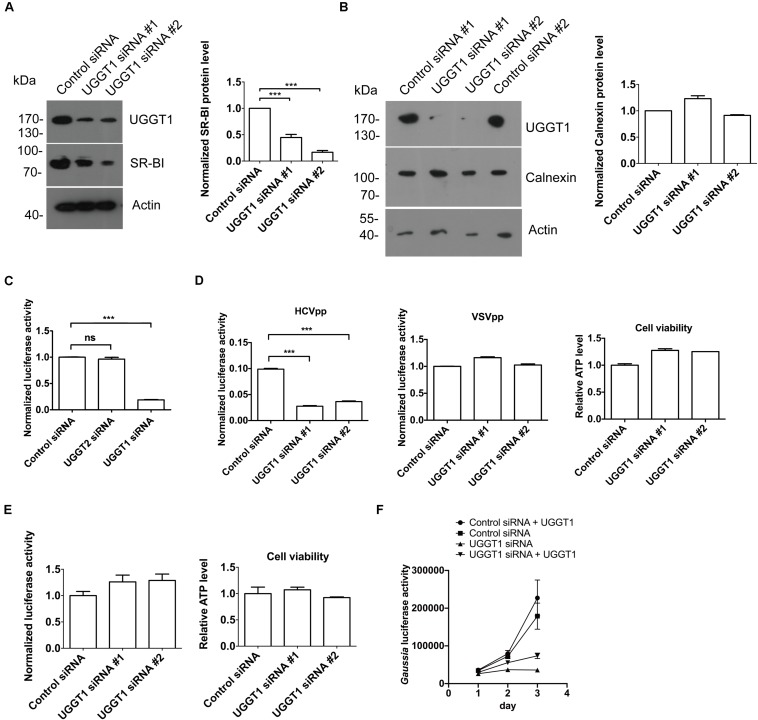
UDP-glucose:glycoprotein glucosyltransferase 1 maintained protein level of SR-BI and promoted HCV entry. **(A)** UGGT1 knockdown decreased SR-BI quantity. Huh7.5.1 cells were treated with two individual UGGT1 siRNAsor control siRNA for 72 h and then cell lysates were subjected to SDS-PAGE followed by Western blotting with indicated antibodies. The blots of three independent experiments were quantified using ImageJ. Relative protein level of SR-BI was normalized to the blot signal of actin. **(B)** Silencing UGGT1 did not change calnexin level. Huh7.5.1 cells were treated with siRNAs against UGGT1 or control siRNAs for 72 h and then cell lysates were subjected to SDS-PAGE followed by Western blotting with indicated antibodies. The blots of three independent experiments were quantified using ImageJ. Relative protein level of calnexin was normalized to the blot signal of actin. **(C)** Silencing UGGT1 reduced HCV infection. Huh7.5.1 cells were treated with siRNAs against UGGT1, UGGT2 or control siRNA for 72 h and then infected with Jc1Flag (p7-nsGluc2A) for another 72 h. The *Gaussia* luciferase activity and ATP levels were measured. The relative luciferase activity was normalized to the cellular ATP levels. Data are presented as the means ± SD. ^∗∗∗^*P* < 0.001; ns, not significant. **(D)** Silencing UGGT1 inhibited HCVpp entry but not VSVpp entry. Huh7.5.1 cells were treated with siRNAs against UGGT1 or control siRNA for 72 h and then infected with HCVpp or VSVpp. The firefly luciferase activity and ATP levels were measured. The relative luciferase activity was normalized to the cellular ATP levels. Data are presented as the means ± SD. ^∗∗∗^*P* < 0.001; ns, not significant. **(E)** UGGT1 knockdown had no effect on established HCV replication. Huh7.5.1 cells were infected with Jc1Flag (p7-nsGluc2A) for 24 h and then treated with siRNAs against UGGT1 or control siRNA for 72 h. The *Gaussia* luciferase activity and ATP levels were measured. The relative luciferase activity was normalized to the cellular ATP levels. **(F)** Overexpression of UGGT1 rescued the HCV infection reduced by UGGT1 siRNA. Huh7.5.1 cells treated with siRNA against UGGT1#2 or control siRNA for 48 h were transfected with UGGT1-Flag construct for another 24 h and then infected with Jc1Flag (p7-nsGluc2A) for another 72 h. The *Gaussia* luciferase activity were measured every 24 h. Data are presented as the means ± SD.

As shown in Figure 4A, silencing UGGT1 with UGGT1 siRNA#1 decreased SR-BI protein level. To further confirm this result, we added another UGGT1 siRNA to perform the knockdown experiment. Both UGGT1 siRNAs reduced SR-BI level compared with control siRNA (Figure 6A). However, calnexin protein levels remained unchanged in the presence of UGGT1 siRNAs, indicating the UGGT1-dependent SR-BI protein quality control was specific (Figure 6B).

Since UGGT1 increased HCV receptor SR-BI protein level, we hypothesized that UGGT1 might promote HCV infection. To test this, we transfected Huh7.5.1 cells with UGGT1 siRNAs and control siRNA for 3 days, and then infected the cells with Jc1Flag (p7-nsGluc2A), an infectious HCV strain, for another 3 days. As shown in Figure 6C, silencing UGGT1 decreased HCV infection. To determine if UGGT1 was required for HCV entry, we silenced UGGT1 by siRNA and examined its effect on HCVpp entry. Knocking down UGGT1 by siRNA inhibited the entry of HCVpp but not VSVpp (Figure 6D). To determine whether UGGT1 was important for established HCV infection, we infected Huh7.5.1 cells with Jc1Flag (p7-nsGluc2A) for 1 day and then silenced UGGT1 by siRNAs for another 3 days. As shown in Figure 6E, silencing UGGT1 had no effect on the established HCV infection. These results demonstrated that UGGT1 was important for HCV entry.

To confirm the specificity of UGGT1 siRNA on HCV infection, we treated Huh7.5.1 cells with siRNA against UGGT1 or control siRNA for 48 h and then transfected UGGT1-Flag or mock for another 24 h. Then the cells were infected with Jc1Flag (p7-nsGluc2A) for 3 days. As shown in Figure 6F, overexpression UGGT1-Flag slightly increased HCV infection accessed by gaussia luciferase activity. UGGT1-Flag overexpression partially restored HCV infection suppressed by UGGT1 siRNA.

### SR-BI Promotes UGGT1-Mediated Protein Folding

**FIGURE 7:**
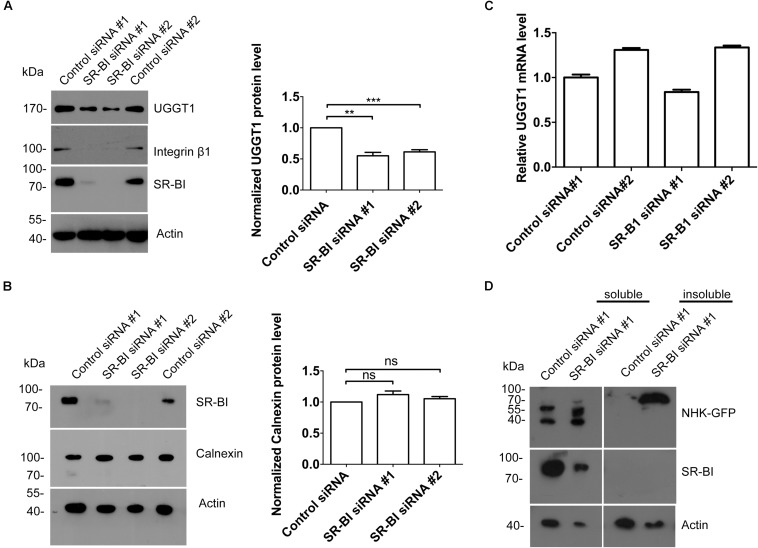
Scavenger receptor class B, type I knockdown decreased protein level of UGGT1 and cellular protein folding activity. **(A)** Silencing SR-BI reduced protein levels of UGGT1 and integrin β1. Huh7.5.1 cells were treated with siRNAs against SR-BI or control siRNAs for 72 h and then cell lysates were subjected to SDS-PAGE followed by Western blotting with indicated antibodies. The blots of three independent experiments were quantified using ImageJ. Relative protein level of UGGT1 was normalized to the blot signal of actin. **(B)** Silencing SR-BI did not change calnexin protein level. Huh7.5.1 cells were treated with siRNAs against SR-BI or control siRNAs for 72 h and then cell lysates were subjected to SDS-PAGE followed by Western blotting with indicated antibodies. The blots of three independent experiments were quantified using ImageJ. Relative protein level of calnexin was normalized to the blot signal of actin. **(C)** Huh7.5.1 cells were treated with siRNA targeting SR-BI or control siRNA for 72 h. Total RNA was isolated and reverse transcribed and then the qPCR was performed. Relative UGGT1 mRNA level was normalized to GAPDH mRNA level. **(D)** SR-BI knockdown inhibited protein folding process of NHK-GFP. 293T cells were treated with siRNAs against SR-BI or control siRNAs for 48 h and then transfected with construct expressing NHK-GFP, a GFP-fused version of the NHK folding variant of 1-antitrypsin for another 24 h. The cells were separated into insoluble portion and soluble portion. Samples were subjected to SDS-PAGE followed by Western blotting with indicated antibodies. ^∗∗^*P* < 0.01, ^∗∗∗^*P* < 0.001.

The interaction between UGGT1 and SR-BI suggested that SR-BI may have an under-appreciated role in N-glycosylated protein quality control. We examined UGGT1 and calnexin abundance during manipulation of SR-BI. Huh7.5.1 cells transfected with SR-BI or control siRNAs for 72 h were analyzed by Western blotting and immunofluorescence microscopy. In the presence of SR-BI siRNA, UGGT1 protein level was reduced while calnexin protein levels remained the same (Figures 7A,B). However, the mRNA level of UGGT1 was not reduced by silencing SR-BI (Figure 7C). UGGT1 functions as a general protein quality sensor, we hypothesized that other N-glycosylated proteins would be influenced by SR-BI knockdown. Indeed, protein level of a well known N-glycosylated protein integrin β1 was reduced by SR-BI siRNA (Figure 7A). Next, we applied a classical UGGT1-mediated protein folding assay using a GFP-fused version of the NHK folding variant of 1-antitrypsin (NHK-GFP). 293T cells treated with SR-BI siRNAs for 72 h were transfected with NHK-GFP plasmid for another 48 h. Cell lysates were separated into insoluble and soluble and accessed by Western blotting analysis. SR-BI siRNA increased insoluble portion of NHK-GFP (Figure 7D). Thus, we concluded that SR-BI promotes UGGT1-mediated protein folding.

## Discussion

UDP-glucose:glycoprotein glucosyltransferase 1 is an ER-resident enzyme that monitors glycoprotein folding by retaining incompletely folded/assembled glycoproteins in the calnexin/calreticulin cycle (Hebert and Molinari, 2012). In this study, we uncovered a role for UGGT1 in N-glycosylation of SR-BI. In the absence of UGGT1, the total level of SR-BI was reduced, and HCV entry was inhibited. Interestingly, SR-BI maintained the protein level of UGGT1 to promote the N-glycosylated protein folding.

N-glycosylation of human SR-BI was validated as the molecular weight was reduced by PNGaseF treatment and mutagenesis. Human SR-BI contains nine sequons for N-linked glycosylation. N to Q mutation of N-glycosylation sites reduced the protein sizes. N-glycosylation inhibitor tunicamycin reduced SR-BI expression level. N-glycosylation deficient SR-BI couldn’t fold properly and ultimately lead to ER-associated degradation. Recently, one point mutations in human SR-BI — at Thr175 (to Ala) — were identified in two subjects with high HDL-C levels (Brunham et al., 2011). This mutation reduced N-glycosylated of SR-BI and HCV infection (Chadwick and Sahoo, 2012; Westhaus et al., 2017). Surprisingly, we did not detect non-glycosylated SR-BI bands (around 55 kDa) in UGGT1-silenced cells or tunicamycin-treated cells. We reasoned that unglycosylated form of SR-BI was not stable and would be degradated.

UDP-glucose:glycoprotein glucosyltransferase 1 is a key component of calnexin cycle in quality control system. As a sensor, UGGT1 ensures that misfolded glycoproteins are recognized by the lectin chaperones and do not leave the secretory pathway (Maattanen et al., 2010). Interestingly, calnexin was also present in SR-BI interactome data set providing additional evidence that SR-BI associates with calnexin cycle. Silencing UGGT1 by siRNA strategy reduced the protein level of SR-BI, indicating the key role of UGGT1 in SR-BI folding. Importantly, HCV entry and infection were impaired in the presence of UGGT1 siRNA. Our data suggested that, UGGT1 added a new layer of control in the SR-BI quality control, providing new insights on the biological role of the N-glycosylation of SR-BI.

About one-third of eukaryotic proteins are destined for the secretory pathway, of which 70% is N-glycosylated. Folding progress of these N-glycosylated proteins is monitored by UGGT1 that selectively recognizes and reglucosylates misfolded and misassembled glycoproteins (Hebert and Molinari, 2012). Previous study found that SR-BI facilitates STAT5 O-glycosylation (Kinslechner et al., 2018). Interestingly, we found a strong association of UGGT1 and SR-BI, which is unique because that not all N-glycosylated protein strongly interacted with UGGT1. We hypothesized that SR-BI might maintain the function of UGGT1 to promote protein folding. Indeed, NHK folding is reduced upon SR-BI silencing. The level of integrin β1 is lower when SR-BI is knocked down. These data suggested that SR-BI played a critical role for N-glycosylated protein folding. SR-BI/UGGT1 interaction might provide a positive feedback for SR-BI N-glycosylation.

SR-BI expression has been linked with during tumor development and malignant progression in many different tumor types (Schorghofer et al., 2015; Gutierrez-Pajares et al., 2016). High level of SR-BI was associated with increased cell migration and motility. SR-BI depletion decreased the migration and invasion of melanoma cells (Kinslechner et al., 2018). Despite these advances, the detailed mechanism how SR-BI contributes cell migration remained to be determined. Our results provided a partial explanation for that. SR-BI maintained the protein level of UGGT1 to enhance the N-glycosylation of integrin β1. Silencing SR-BI reduced the protein level of integrin β1, which is important for cell migration and tumor development.

In this study, elucidating the SR-BI interactome identified the interaction between SR-BI and calnexin cycle components by affinity precipitation coupled with MS. UGGT1 played a key role for SR-BI protein quality control. Moreover, UGGT1 promoted HCV entry. Interestingly, SR-BI is important for UGGT1 mediated N-glycosylated protein folding. Thus, SR-BI/UGGT1 interaction played key roles for glycoproteins quality control. The central role for UGGT1 in SR-BI ER quality control machinery further makes it a potential pharmacological target in SR-BI related pathological conditions including HCV infection. Our findings will set up the basis for studying the more function of SR-BI.

## Data Availability

All datasets generated for this study are included in the manuscript and/or the Supplementary Files.

## Author Contributions

LZ contributed to supervision, conceptualization, and funding acquisition for this study. JH, HY, PY, XJ, SS, and JL investigated the study. JH performed the methodology. JH and LZ contributed to writing the original draft, review, and editing the manuscript.

## Conflict of Interest Statement

The authors declare that the research was conducted in the absence of any commercial or financial relationships that could be construed as a potential conflict of interest.

## Footnotes

1 http://www.cbs.dtu.dk/services/NetNGlyc/
