# Supplementary material for: SR-BI Interactome Analysis Reveals a Proviral Role for UGGT1 in Hepatitis C Virus Entry
Source: Front Microbiol. 2019 Sep 6;10:2043. doi: 10.3389/fmicb.2019.02043 (PMC6743029; doi:10.3389/fmicb.2019.02043)
Supplement: Supplementary file 1 [file Table_1.DOCX]

Supplemental Table 1. Potential binding partners of SR-BI in IP/MS identification.

| **Protein Access Number** | **Protein Description** | **Name** | **Score** | **MatchedPeptides** |
| --- | --- | --- | --- | --- |
| Q9NYU2 | UDP-glucose:glycoproteinglucosyltransferase 1 | UGGT1 | 5072 | 216 |
| P78527 | DNA-dependent protein kinase catalytic subunit | PRKDC | 3087 | 210 |
| P27824 | Calnexin | CANX | 2678 | 117 |
| O14980 | Exportin-1 | XPO1 | 1398 | 71 |
| Q00610 | Clathrin heavy chain 1 | CLTC | 1328 | 69 |
| P05023 | Sodium/potassium-transporting ATPase subunit alpha-1 | ATP1A1 | 1204 | 55 |
| P55060 | Exportin-2 | CSE1L | 1137 | 58 |
| Q14697 | Neutral alpha-glucosidase AB | GANAB | 1110 | 63 |
| O43592 | Exportin-T | XPOT | 1023 | 44 |
| P04843 | Dolichyl-diphosphooligosaccharide--protein glycosyltransferase subunit 1 | RPN1 | 891 | 44 |
| P04844 | Dolichyl-diphosphooligosaccharide--protein glycosyltransferase subunit 2 | RPN2 | 852 | 34 |
| Q92616 | eIF-2-alpha kinase activator GCN1 | GCN1 | 829 | 72 |
| Q8NEZ5 | F-box only protein 22 | FBXO22 | 818 | 28 |
| P05141 | ADP/ATP translocase 2 | SLC25A5 | 798 | 52 |
| O00410 | Importin-5 | IPO5 | 762 | 44 |
| P14618 | Pyruvate kinase PKM | PKM | 723 | 29 |
| P42704 | Leucine-rich PPR motif-containing protein, mitochondrial | LRPPRC | 722 | 45 |
| P16615 | Sarcoplasmic/endoplasmic reticulum calcium ATPase 2 | ATP2A2 | 683 | 45 |
| Q14974 | Importin subunit beta-1 | KPNB1 | 674 | 30 |
| P35232 | Prohibitin | PHB | 667 | 27 |
| P68104 | Elongation factor 1-alpha 1 | EEF1A1 | 660 | 42 |
| P02768 | Serum albumin | ALB | 592 | 42 |
| P13637 | Sodium/potassium-transporting ATPase subunit alpha-3 | ATP1A3 | 582 | 31 |
| P50993 | Sodium/potassium-transporting ATPase subunit alpha-2 | ATP1A2 | 530 | 27 |
| P19338 | Nucleolin | HSP90B1 | 526 | 28 |
| P14625 | Endoplasmin | NCL | 519 | 29 |
| Q99623 | Prohibitin-2 | PHB2 | 517 | 28 |
| Q96P70 | Importin-9 | IPO9 | 514 | 24 |
